# Transcriptomics reveals extensive inducible biotransformation in the soil-dwelling invertebrate *Folsomia candida *exposed to phenanthrene

**DOI:** 10.1186/1471-2164-10-236

**Published:** 2009-05-20

**Authors:** Benjamin Nota, Mirte Bosse, Bauke Ylstra, Nico M van Straalen, Dick Roelofs

**Affiliations:** 1 VU University Amsterdam, Institute of Ecological Science, Department of Animal Ecology, De Boelelaan 1085, 1081 HV, Amsterdam, The Netherlands; 2 VU University Medical Center, Department of Pathology, De Boelelaan 1117, 1081 HV, Amsterdam, The Netherlands

## Abstract

**Background:**

Polycyclic aromatic hydrocarbons are common pollutants in soil, have negative effects on soil ecosystems, and are potentially carcinogenic. The Springtail (Collembola) *Folsomia candida *is often used as an indicator species for soil toxicity. Here we report a toxicogenomic study that translates the ecological effects of the polycyclic aromatic hydrocarbon phenanthrene in soil to the early transcriptomic responses in *Folsomia candida*.

**Results:**

Microarrays were used to examine two different exposure concentrations of phenanthrene, namely the EC_10 _(24.95 mg kg^-1 ^soil) and EC_50 _(45.80 mg kg^-1 ^soil) on reproduction of this springtail, which evoked 405 and 251 differentially expressed transcripts, respectively. Fifty transcripts were differential in response to either concentration. Many transcripts encoding xenobiotic detoxification and biotransformation enzymes (phases I, II, and III) were upregulated in response to either concentration. Furthermore, indications of general and oxidative stress were found in response to phenanthrene. Chitin metabolism appeared to be disrupted particularly at the low concentration, and protein translation appeared suppressed at the high concentration of phenanthrene; most likely in order to reallocate energy budgets for the detoxification process. Finally, an immune response was evoked especially in response to the high effect concentration, which was also described in a previous transcriptomic study using the same effect concentration (EC_50_) of cadmium.

**Conclusion:**

Our study provides new insights in the molecular mode of action of the important polluting class of polycyclic aromatic hydrocarbons in soil animals. Furthermore, we present a fast, sensitive, and specific soil toxicity test which enhances traditional tests and may help to improve current environmental risk assessments and monitoring of potentially polluted sites.

## Background

Polycyclic aromatic hydrocarbons (PAHs) are a common source of pollution in soil, mostly caused by anthropogenic means. PAHs can be derived from incomplete combustion or fossil fuel processing, and the highest concentrations in the environment are found in urban areas [1]. Several PAHs are known carcinogens [2,3], which makes this class of pollutants not only hazardous to the environment, but also to human health. Within many, if not all, organisms, detoxification of xenobiotics like PAHs can be divided in three phases. In phase I toxic compounds are modified resulting in more reactive metabolites. The best known enzymes involved in phase I are the cytochrome P450s [4]. In the second phase the reactive metabolites are conjugated with chemical groups like glutathione or glucuronic acid [3,5]. These conjugation reactions are performed by enzymes known as transferases. In phase III specialized transporters recognize the conjugates, and expel them out of the cell [6].

Springtails (Collembola) are soil-dwelling arthropods, and are therefore most suitable for soil toxicity testing. They have a detritivorous role in the soil ecosystem, i.e., they contribute to decomposition and recycling of nutrients within soil. Springtails are most abundant in soil and are often reported to be the most sensitive to pollution [7], and particularly to PAHs [8]. The Springtail *Folsomia candida *is often used in standardized ecotoxicity testing of soil [7], e.g., the International Organization for Standardization (ISO) test 11267 [9]. In these standardized tests the effect on reproduction is examined after exposure to contaminated soil for 28 days. New molecular techniques like genomics (e.g., microarrays) have been proposed to enhance environmental toxicity tests [10]. Genomics could help make existing standardized tests: faster, more specific, and more sensitive [11]. *F. candida*'s transcriptome is partially sequenced and available in Collembase [12,13], which makes this animal suitable for soil toxicogenomic studies. A previous toxicogenomic study with *F. candida *in cadmium contaminated soil revealed that gene expression profiles indicate toxicity already within 2 days [14]. Another toxicogenomic study in crustaceans further demonstrated that chronic consequences of environmental stress on populations could be predicted from early changes in gene expression [15].

In the present study we evaluated the transcriptomic response of *F. candida *in PAH contaminated soil. We used the compound phenanthrene as a model for PAHs, and spiked field soil (LUFA 2.2) with two different concentrations. The concentrations used in this study had different effects on reproduction, after 28 days. We used phenanthrene concentrations of 24.95 and 45.80 mg kg^-1 ^soil, which represents the EC_10 _(10% reduction) and EC_50 _(50% reduction) on reproduction, respectively [16]. Although, such high concentrations of single PAH compounds have not been reported in the environment, the sum of 15 PAHs (including phenanthrene) was reported to be higher than these concentrations in several European cities [1]. Our aim was to elucidate the early molecular response to-, and the toxic mechanism of PAHs in *F. candida *with the use of transcriptomic analysis. The potential of this technique to improve terrestrial and springtail ecotoxicology is also evaluated. Furthermore, we examined whether the different concentration effects on reproduction could be explained by the transcriptomic response. To our knowledge this is the first transcriptomic study of phenanthrene toxicity in non-mammalian animals.

## Results and Discussion

**Figure 1:**
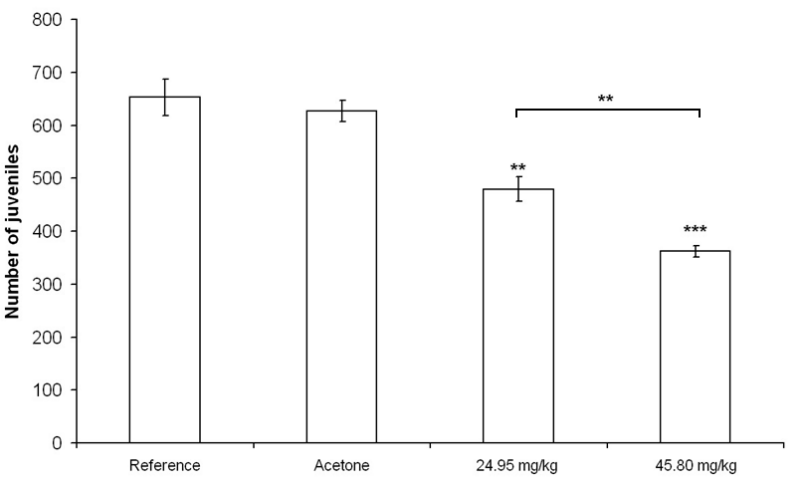
**Effect of phenanthrene on reproduction**. The average (n = 5) number of *Folsomia candida *juveniles per jar after 28 days exposure to reference, and solvent (acetone) control soil, and soil containing 24.95 mg kg^-1^, and 45.80 mg kg^-1 ^phenanthrene. The acetone treatment did not significantly affect (t-test) reproduction compared to the reference soil, but both phenanthrene concentrations did significantly effect reproduction indicated by asterisks: **P < 0.01; ***P < 0.001. Error bars indicate standard errors.

Many uncontrolled factors can cause variability in results derived from soil toxicity tests with *Folsomia candida *[17], which may affect reproducibility. To verify that the phenanthrene concentrations in soil, taken from the literature [16], had a significant and reproducible effect on the reproduction of *F. candida *in our experiment, we performed a 28 days exposure toxicity test. Nominal phenanthrene concentrations of 24.95 (EC_10_) and 45.80 (EC_50_) mg kg^-1 ^soil, and also a solvent (acetone) control were tested. Clean LUFA 2.2 soil was used as reference (untreated control). The solvent control did not show a significant effect on reproduction compared to clean reference LUFA 2.2 soil, but phenanthrene concentrations of 24.95 and 45.80 mg kg^-1 ^soil did have significant effects (Figure 1). Reproduction was reduced with 27% and 45%, respectively, compared to the reference soil. Actual phenanthrene concentrations in soil were measured using high performance liquid chromatography at the start and the end (28 days) of the experiment. The concentrations of phenanthrene in soil after 28 days were approximately 60% of the initial concentrations, which is expected for non-persistent organic compounds like phenanthrene.

To identify transcripts that responded to the phenanthrene exposure, we used gene expression microarray analysis. Microarrays were constructed containing 5,069 different 60 nt long DNA probes, printed in triplicate. The probe sequences were taken from an earlier array design, described in a previous study [14]; each probe represents a different gene cluster from Collembase [12,13]. Adult animals were exposed for 2 days to clean LUFA 2.2 soil (reference), and soil spiked with nominal phenanthrene concentrations of 24.95 and 45.80 mg kg^-1 ^soil. No solvent control was used in the gene expression experiment, because it did not show a significant effect on reproduction. However, it cannot be excluded that acetone had a minor effect on gene expression. RNA was extracted from all animals from each experimental jar, constituting one biological replicate sample. We applied a so called replicated reference design [18]; every treated sample was hybridized to a unique reference (untreated) sample. This resulted in 8 reference, 4 EC_10 _treated, and 4 EC_50 _treated samples. To assess significant differential transcripts, linear models and empirical Bayes statistics were used [19]. Derived *p*-values were corrected for multiple testing using Benjamini-Hochberg's (BH) method [20].

We identified 405 and 251 differentially expressed transcripts (BH corrected *p *< 0.05), following exposure to phenanthrene concentrations of 24.95 and 45.80 mg kg^-1 ^soil, respectively. Remarkably, only 50 transcripts were differentially expressed at both concentrations (Additional file 1: Figure S1). Many transcripts of *F. candida *could not be annotated and have therefore an unknown function. Future sequencing efforts, however, are on their way and hopefully more transcripts can be annotated in the near future.

### Exposure to low effect concentration of phenanthrene

Of the 405 transcripts that were significantly differential in response to the low effect concentration of phenanthrene (24.95 mg kg^-1 ^soil), 260 transcripts were upregulated and 145 transcripts were downregulated compared to the reference. All differentially expressed transcripts that responded to the low concentration of phenanthrene are available in Additional file 2: Table S1. Their putative function is based on sequence homology (e.g., BLAST, interPro). Seven transcripts encoding cytochrome P450s were upregulated. Cytochrome P450 enzymes are most commonly involved in monooxygenase reactions [4]. In addition to this, we also identified other monooxygenases being up- and downregulated in response to the low concentration of phenanthrene. The upregulated cytochrome P450s and other monooxygenases are most likely involved in phase I of the biotransformation and detoxification of phenanthrene. Furthermore, we found upregulation of aldehyde oxidases, carboxylesterases, and short-chain dehydrogenases, which are probably also involved in phase I reactions with phenanthrene [5,21,22]. Many transferase enzymes are involved in phase II of the biotransformation of xenobiotics. In this phase the reactive metabolites created in phase I are being conjugated with polar groups like glutathione or sugar groups [5]. We identified 7 transcripts encoding glutathione S-transferases upregulated in response to the low concentration of phenanthrene, and one was downregulated. Also many transcripts encoding proteins that contain a UDP-glucuronosyl/UDP-glucosyltransferase domain were upregulated. Stroomberg et al. showed that the phase II biotransformation of the PAH pyrene in *F. candida *produced the metabolite pyrene-1-glucoside, but not pyrene-1-glucuronide [23]. Therefore we assume that these induced transcripts actually encode UDP-glucosyltransferases, and not UDP-glucuronosyltransferases. Membrane transporters which are involved in phase III were also significantly upregulated; we identified 3 ABC-transporters.

The low concentration of phenanthrene induced 2 transcripts encoding heat shock proteins (HSPs) and one chaperonin which are likely part of the general stress response. Many transcripts encoding ribosomal proteins and a few translation initiation factors were also upregulated, which indicates increased protein translation. The translation of all the biotransformation enzymes might be a reason for this increase. Interestingly, many transcripts encoding chitin binding proteins and chitinases were also upregulated. These gene products, together with the upregulation of a transcript with homology to the molting fluid carboxypeptidase A [24], could be involved in the molting process or the formation of the peritrophic envelope. This peritrophic envelope is excreted by the gut epithelial cells in most arthropods, and is a thin membrane which has protective functions against abrasive food particles, invading pathogens, plant toxins, and oxidative damage [25]. Phase I metabolites of PAHs can often generate reactive oxygen species (ROS) and can cause oxidative damage. In humans, the microbiota in the colon was able to bioactivate PAHs [26], and we therefore suggest that ingested phenanthrene is being transformed to ROS forming metabolites by the microbiota in *F. candida*'s gut. The peritrophic envelope could then function as an antioxidant to protect the epithelial gut cells from ROS. However, further research is needed to confirm ROS production by the microbiota in *F. candida*'s gut. Endogenous transformation of PAHs by cytochrome P450s also generates ROS, and we found transcripts encoding superoxide dismutase (copper/zinc binding) and catalase both upregulated in response to the low concentration of phenanthrene.

Other transcripts that are worth mentioning are vitellogenin and genes containing a vitelline membrane outer layer protein I (VOMI) domain. These transcripts, all upregulated, are involved in egg production. This suggests that phenanthrene is disrupting the reproduction process in *F. candida *in a direct manner. Also many transcripts encoding proteins containing a zinc finger domain were significantly affected (up- or downregulated). Zinc finger domains are often involved in DNA binding, like for example in transcription factors [27]. Most of these transcripts were downregulated, which suggests that many, perhaps less essential, processes were switched off in order to focus on more essential transcripts that cope with phenanthrene detoxification. Furthermore, transcripts involved in post-transcriptional modifications of RNA, or post-translational modifications of proteins were also affected. Post-translational modification is possibly an indication of altered signal transduction. For example, genes from the RAS family were downregulated. It is however difficult to predict exactly which processes (e.g., cell proliferation or apoptosis) are influenced by these signal transduction pathways, but it could suggest carcinogenic potential of phenanthrene in higher animals.

### Exposure to high effect concentration of phenanthrene

Compared to the reference, 251 transcripts were significantly differentially expressed in response to the high effect concentration of phenanthrene (45.80 mg kg^-1 ^soil), 122 transcripts were upregulated and 129 transcripts were downregulated. This is clearly less than in the low concentration exposure. A difference with the low concentration exposure is that we here observed relatively more genes downregulated than upregulated. All differentially expressed transcripts that responded to the high concentration of phenanthrene are available in Additional file 3: Table S2. We can again identify transcripts involved in all three biotransformation and detoxification phases. For phase I, we observed upregulation of 4 cytochrome P450s and one NADPH cytochrome P450 reductase, and many other differentially expressed monooxygenases (up- and downregulated). Furthermore, we observed upregulated carboxylesterases, and short-chain dehydrogenases, which are likely also involved in phase I reactions. Transcripts encoding glutathione S-transferases and UDP-glucuronosyl/UDP-glucosyltransferase domain containing proteins were also upregulated, which are probably involved in phase II reactions. Six transcripts encoding ABC-transporters were also upregulated; they could be involved in phase III. This is twice the amount of transporters compared to the low concentration of phenanthrene exposure.

The HSP genes that were upregulated by the low concentration of phenanthrene were also upregulated by the high concentration of phenanthrene, which is indicative for general stress. Also here one chaperonin was induced, but interestingly, it was not the same transcript that was induced in response to the low concentration. Furthermore, one transcript containing a DnaJ (HSP40) domain was downregulated in response to the high concentration. A few transcripts encoding ribosomal proteins were all downregulated, which indicate a suppression of protein translation, although a translation initiation factor and a tRNA synthetase were both upregulated. The suppression of these ribosomal proteins is a clear difference between the low and high concentration phenanthrene exposure, because the low concentration induced several other ribosomal proteins. The synthesis of proteins is an energy costly process, and is therefore often suppressed in stressful situations like in detoxification of xenobiotics, in order to reallocate energy budgets [28]. Furthermore, we identified upregulated transcripts involved in the synthesis of the antibiotic compound penicillin, and downregulated C-type lectins. This suggests that the high concentration of phenanthrene is evoking an immune response, and thus increases susceptibility to pathogens. We observed upregulation of a superoxide dismutase (copper/zinc binding), indicating oxidative stress. This gene was, however, not the same superoxide dismutase that was upregulated in response to the low concentration of phenanthrene. Nevertheless, other transcripts, like glutaredoxins and thioredoxins, that play a role in oxidative stress, were downregulated. More transcripts that were affected in response to the high effect concentration of phenanthrene noteworthy to mention were involved in: transcription and chromatin remodeling, DNA replication, post-transcriptional and post-translational processes, and signal transduction.

### Comparison between low and high exposures

**Figure 2:**
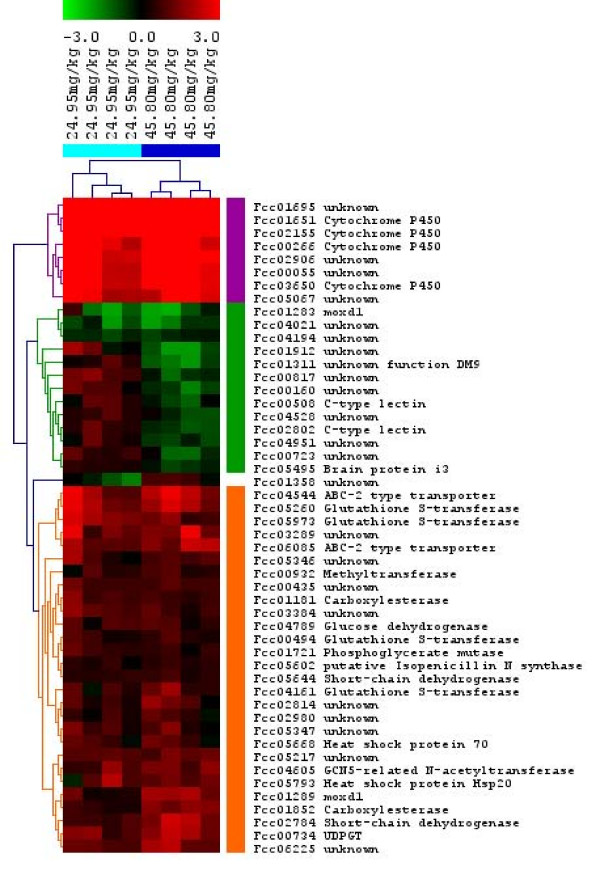
**Heatmap for transcripts differentially expressed in response to each of the phenanthrene concentrations**. Four microarrays were used for each exposure concentration. Hierarchical clustering was performed using log_2 _fold change values (treatment/reference). Red indicates upregulation and green downregulation compared to the reference, black indicates no difference. The transcripts are named by their gene cluster in Collembase, followed by their predicted function.

Only 50 transcripts were differentially expressed in response to both exposure concentrations. We used hierarchical clustering to group the similarly expressed transcripts (Figure 2). The transcripts can roughly be divided into three separate groups. The first group contains transcripts that were highly upregulated in response to both phenanthrene concentrations (most upper [purple] group in Figure 2). This group contains cytochrome P450s and unknown genes (with no significant homology). The second group created by hierarchical clustering contains transcripts that were moderately upregulated in response to both phenanthrene concentrations (lower [orange] group in Figure 2). In this group we indentified transcripts encoding e.g., heat shock proteins, glutathione S-transferases, and ABC-transporters. The third group (middle [green] group in Figure 2) contains transcripts which were all downregulated in response to the high concentration of phenanthrene. In response to the low concentration, some transcripts were also downregulated in this group, but most transcripts, including C-type lectins, were slightly upregulated.

Of the 50 transcripts, we wanted to identify which responded different between the two phenanthrene concentrations. The transcripts that differ significantly could help to explain the different effects on reproduction after 28 days exposure. These transcripts that responded differently between the two concentrations were identified using a t-test, and 16 were found to respond significantly different (*p*-value < 0.05) between the two concentrations (see Table 1). Many of the genes have an unknown or unclear function. Interestingly, the expression of genes encoding proteins with possible detoxifying and biotransformational functions, (carboxylesterase, monooxygenase, short-chain dehydrogenase, and cytochrome P450), all increased with increasing phenanthrene concentration. This implies that these genes responded in a concentration dependent manner, and are likely good quantitative biomarkers for the relevant phenanthrene concentrations in soil. Two C-type lectins, which are assumed to play a role in the invertebrate immune system [29], are upregulated in response to the low, but downregulated in response to the high concentration of phenanthrene. This suggests again, like already mentioned above, that the high concentration of phenanthrene had a negative impact on the immune system. This seems to be likely, because more genes involved in the production of antibiotics responded to the high concentration. An impaired immune response was observed in an earlier toxicogenomic study with *F. candida *exposed to cadmium [14]. This implies that the immune response is correlated with suppression of reproduction, no matter the mode-of-action of the toxicant. Interestingly, a recent toxicogenomic study with earthworms, wherein *Eisenia fetida *was exposed to soil containing 2,4,6-trinitrotoluene [30], also showed an impaired immune response.

**Table 1:** Transcripts with a significant difference in gene expression between two phenanthrene exposures

Gene cluster^a^	Putative function	Fold change (24.95 mg/kg)^b^	Fold change (45.80 mg/kg)^c^	*p*-value^d^
Fcc01852	Carboxylesterase	1.41	2.84	0.0008
Fcc01695	unknown	13.65	35.91	0.0013
Fcc00817	unknown	2.24	-1.52	0.0014
Fcc01311	Protein of unknown function DM9	1.44	-2.36	0.0014
Fcc02802	C-type lectin	1.59	-1.64	0.0018
Fcc00160	unknown	1.80	-1.80	0.0038
Fcc05495	brain protein i3	1.33	-1.38	0.0048
Fcc04528	unknown	1.55	-1.44	0.0103
Fcc01289	monooxygenase	1.46	3.41	0.0107
Fcc00723	unknown	1.64	-1.63	0.0144
Fcc01912	unknown	1.70	-2.54	0.0194
Fcc00508	C-type lectin	1.56	-1.46	0.0218
Fcc01358	unknown	-1.63	1.50	0.0221
Fcc04951	unknown	1.39	-1.56	0.0226
Fcc02784	Short-chain dehydrogenase	1.67	3.54	0.0288
Fcc01651	Cytochrome P450	12.12	19.72	0.0298

^a^Gene cluster as can be found in Collembase, ^b^fold change in response to 24.95 mg/kg phenanthrene in soil compared to reference soil, ^c^fold change in response to 45.80 mg/kg phenanthrene in soil compared to reference soil, ^d^*p*-value derived with t-test (n = 4).

Many transcripts were differentially expressed only in response to one phenanthrene concentration, but shared similar putative functions. For instance, we found ABC-transporters that were only significantly upregulated in response to the low concentration, but not to the high concentration of phenanthrene, and vice versa. Other examples are the already above mentioned superoxide dismutases, and chaperonins. Interestingly, the transcripts involved in all three phases of the detoxifying and biotransformation of xenobiotics were upregulated in response to both phenanthrene concentrations. However, only a few were differentially expressed in response to both concentrations, and then only a few of them were regulated in a concentration responsive manner. This demonstrates the complexity of the transcriptional regulation of these biotransformation enzymes. The transcripts involved in chitin metabolism were mostly upregulated in response to the low concentration of phenanthrene. An explanation might be that the high phenanthrene concentration could kill or inhibit the ROS forming microorganisms in the gut. Therefore, the synthesis of a peritrophic envelope would be less necessary.

The larger number of differentially expressed genes in response to the low concentration of phenanthrene, compared with the high concentration, was likely caused by the reallocation and distribution of the animal's energy budget. A higher concentration of phenanthrene in the organism would switch priorities to the production of biotransformation enzymes, and would leave less energy left for regulation of other less essential cellular processes and e.g., reproduction. Especially the genes encoding the biotransformation enzymes that responded in a concentration dependent manner indicate the importance of this process in order to cope with phenanthrene toxicity.

### Comparison with the cadmium induced transcriptome

Compared to our previous transcriptomic study [14], (data available under GEO [31] accession number GSE11122), wherein *F. candida *was also exposed for two days to cadmium polluted soil (also EC_50 _on reproduction after 28 days), the number of genes that responded are by far less. After 2 days of exposure to cadmium (EC_50_) polluted soil, a total of 964 differentially expressed transcripts were identified compared to 251 for phenanthrene (EC_50_) polluted soil. One hundred and twelve transcripts are significantly differentially expressed in response to both compounds, which is almost half of the phenanthrene responsive transcripts. Although the experimental conditions were similar in both exposure experiments, we have to be cautious comparing these two datasets, because the two microarray designs were technically slightly different. In the present study a custom Agilent microarray with an 8 × 15 k format was used and in the previous (cadmium) study we used a custom Agilent microarray with a 2 × 11 k format. Consequently, the spots on the two different microarrays varied in diameter. Furthermore, the microarray in this study contained 5,069 different probes printed in triplicate and the microarray in the previous study contained 5,131 different probes printed in duplicate. However, Shi et al. (2006) showed high consistency across different microarray platforms [32], supporting data comparison between different platforms to some extent. Thus, if we compare the results of the present phenanthrene exposure study with the results of our previous cadmium exposure study, we can see that many transcripts respond in the same manner and other transcripts respond differently. The transcripts that respond similar to both compounds with similar effect concentrations are potential biomarkers for level (degree) of soil toxicity, and transcripts that respond differently are potential compound specific biomarkers. In accordance with our results, this was also shown in previous toxicogenomic studies in e.g., yeast [33], where a general environmental stress response can be distinguished from a treatment specific stress response. In Additional file 4: Figure S2 a heat map is shown of hierarchical clustering of the expression of all significant transcripts in response to both compounds. Transcripts encoding monooxygenase (Fcc01289) or short-chain dehydrogenase (Fcc02784) were upregulated in response to both compounds. These transcripts were significantly lower expressed in response to the low concentration of phenanthrene. It seems that their transcription is more regulated by the toxic effect concentration, but independent of which xenobiotic compound is used. Such biomarkers would be very useful for fast toxicity screening of potentially polluted sites. Further research is, however, needed to validate the usefulness of these transcripts as biomarkers.

### Quantitative RT PCR (qPCR) validation

**Figure 3:**
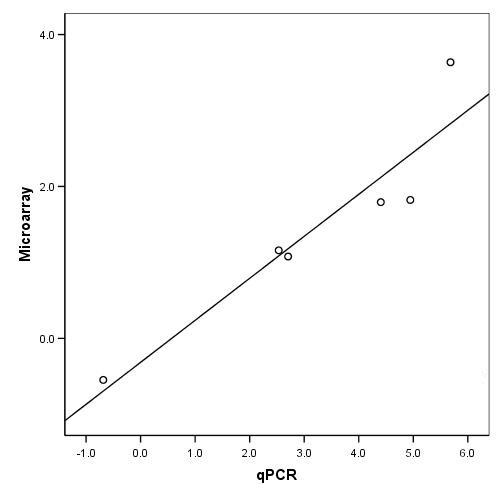
**Correlation between gene expressions measured in *Folsomia candida *using microarray analysis and qPCR**. The average log_2 _fold change values were used, and each point represents a differentially expressed gene. A significant correlation of 0.943 (Spearman's Rho, *p *< 0.01) was found.

**Figure 4:**
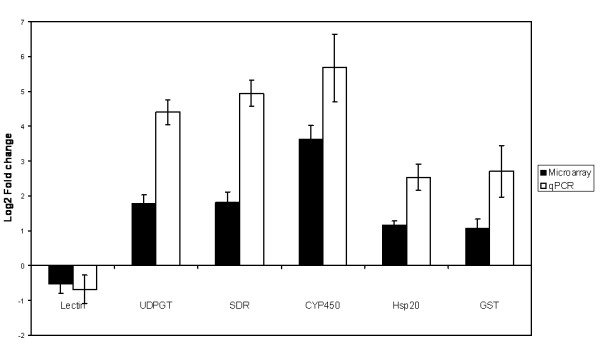
**Comparison between gene expression derived from microarray analysis and qPCR**. In the histogram the average log_2 _fold change is shown for 6 different genes, in response to the high effect concentration of phenanthrene (45.80 mg kg^-1 ^soil). The genes used are from left to right: Fcc00508 (C-type lectin), Fcc00734 (UDP-glucosyltransferase), Fcc02784 (short-chain dehydrogenase), Fcc03650 (cytochrome P450), Fcc05793 (heat shock protein), and Fcc05973 (glutathione S-transferase). Error bars indicate standard errors.

To validate our microarray platform we selected 6 genes with different functions and performed quantitative RT PCR (qPCR). We used the same RNA samples (treated and untreated) that were used in the high effect concentration phenanthrene exposure microarray experiment. *YWHAZ *was used as reference gene for normalization, because it was shown to be one of the most stable endogenous genes in *F. candida *(de Boer et al., in press [34]). First, the log_2 _transformed fold change was calculated for the same pairs of RNA samples (treated vs. untreated) that were hybridized on the microarrays. Then, the average log_2 _fold change was calculated for each transcript and a significant correlation of 0.943 (Spearman's Rho, *p *< 0.01) was found between the qPCR and microarray platforms (Figure 3). We can observe that the log_2 _transformed fold change values are reduced in the microarray data compared to the qPCR (Figure 4). This is often observed between microarray and qPCR data and is probably due to loess normalization [35].

## Conclusion

This first transcriptomic study on phenanthrene toxicity in an invertebrate showed some remarkable results. First, the number of significantly affected genes decreased with increasing effect concentration. Second, the low effect concentration showed more up- than downregulated genes relative to the high effect concentration. Third, significant genes shared by both treatments showed a clear signature of biotransformation of phenanthrene. This demonstrates that *F. candida *has comparative responses to other organisms and is therefore a representative model organism for soil toxicogenomics. We have shown a potentially fast method to evaluate soil toxicity on the molecular level, in an ecologically relevant species, and validated our results with a different technique. Furthermore, the method is sensitive and discriminative between different toxic compounds. In this study insight was gained not only about the early toxic mode of action of PAH in soil on Collembola, but the method may also further be used to gain insight on other polluting (new) compounds, which will be beneficial for environmental risk assessment studies. Besides that, this method promises potential to be used for fast screening and monitoring of potentially polluted sites. In this way a fast pre-selection can be made in order to identify the most severe polluted sites, which then should receive the highest priority for further ecotoxicity testing and remediation. Together with similar new toxicogenomic methods in other soil-dwelling animals like earthworms [30,36-38], this approach may potentially change the current field of terrestrial ecotoxicology.

## Methods

### Phenanthrene exposure experiments

The collembolan *Folsomia candida *('Berlin strain'; VU University Amsterdam) was used for the exposure experiments. Cultures were first age synchronized before exposure to soil, following standardized methods [9]. In short, 20 adult animals were incubated on plastic rings; containing plaster of Paris mixed with charcoal, to lay eggs and were removed after 2 days. The incubation conditions were 20°C with a 12 hour light/dark cycle. It took approximately 10 days before the eggs hatched. All exposure experiments were done using a standardized natural soil (LUFA 2.2, Spreyer, Germany). Soil was spiked with two different phenanthrene (98% purity, Sigma-Aldrich) concentrations (24.95 and 45.80 mg kg^-1 ^soil). Phenanthrene was added to the soil in an acetone (Riedel-de Haën) solution. Equal volumes of acetone were used for spiking, and it was applied to 10% of the soil. The phenanthrene/acetone solutions covered the soil completely, and were left together with the soil for 24 hours in a closed glass container to incubate. Then the glass containers were opened for 24 hours in order to let the acetone evaporate, and the rest of the soil (90%) was added before moisturizing. The moisture levels of the soil were ~22% (w/w), which is 50% of the water holding capacity. The prepared soil was kept at 4°C, and was used for exposure experiments within a week. Controls (non-spiked reference soil and solvent treated soil) were also included, and all exposure experiments were done at 20°C with a 12 hour light/dark cycle.

To verify that the phenanthrene concentrations of 24.95 and 45.80 mg kg^-1 ^soil had a significant effect on reproduction, ten synchronized juveniles (10 d old) were exposed to ~30 grams of non-spiked (reference), solvent (acetone) control, or phenanthrene spiked soil in 100 mL glass jars, using 5 replicates (jars) for each treatment. The jars were opened for aeration twice a week, when needed the moisture levels were adjusted using demineralized water. Once a week they were fed with baker's yeast (Dr. Oetker), following as much as possible the standardized guide lines [9]. After 28 d exposure the jars were filled with 100 mL tap water and emptied in a glass beaker, through which the animals were extracted from the soil by means of floatation. The floating animals were photographed, and juveniles were counted using cell^D ^software (Olympus).

To examine gene expression in *F. candida *exposed to phenanthrene spiked soil, first again age synchronized cultures were obtained. Thirty animals (23 d old) were exposed per jar containing ~30 grams of non-spiked or phenanthrene (24.95 and 45.80 mg kg^-1^) spiked soil for 2 days, but now using 4 replicates per phenanthrene treatment and 8 replicates for the non-spiked soil exposure treatment. Animals were extracted from soil by means of floatation with 100 mL tap water, and then scooped from the water surface using a small spoon and put first on plastic rings containing plaster of Paris mixed with charcoal to remove surplus water. The animals were then taken from the rings and were put in microcentrifuge tubes in which they were snap frozen in liquid nitrogen. The whole harvesting procedure from scooping to freezing took approximately 5 minutes per sample.

### Measurement of phenanthrene in soil samples

Actual phenanthrene concentrations in soil were determined at the beginning and end of the 28 days exposure experiment. For this purpose approximately 1 g of moist soil was mixed with equal amount of anhydrous sodium sulfate and Soxhlet extracted for 5 h using hexane. The samples were then transferred to acetonitrile using a gentle flow of nitrogen. Samples were measured using a high performance liquid chromatography (HPLC) system consisting of a Vydac RP 18 201TP column with a Vydac 201 GD RP-18 guard column (Alltech), a Jasco FP-1520 fluorescence detector (Jasco), and a Gynkotek UVD320s ultraviolet diode-array detector (Gynkotek).

### RNA preparation, amplification, labeling and hybridization

Each biological replicate sample consisted of total RNA extracted from 30 animals exposed in the same jar to either non-spiked (reference) or phenanthrene spiked soil. Total RNA was extracted from the samples using the SV Total RNA Isolation system (Promega), which included a DNase treatment. 500 ng input of total RNA was used for amplification and labeling with the Agilent Low-Input Fluorescent Linear Amplification Kit (Agilent Technologies), according to the manufacturer's guidelines with a slight modification where the transcription (labeling) reactions were done in half the volume. Labeled and amplified cRNA was purified using RNeasy (Qiagen), and the Agilent Gene Expression Hybridization Kit (Agilent Technologies) was used for hybridization, which was done at 65°C for 17 h rotating at 10 rpm in an incubator. For hybridization a so called replicated reference design was used, wherein the 4 low phenanthrene concentration (24.95 mg kg^-1^) exposed samples and the 4 high phenanthrene concentration (45.80 mg kg^-1^) exposed samples were mixed with 8 unique non-spiked reference samples. For each phenanthrene concentration or reference, half of the samples were labeled with the fluorescent dye Cy3 and the other half with Cy5. The total microarray experiment resulted in 8 hybridizations of 16 unique samples. After hybridization, the arrays were washed using Gene Expression Wash Buffer Kit (Agilent Technologies), and scanned with the default settings on an Agilent DNA microarray scanner (Agilent Technologies).

For this experiment Custom Gene Expression Microarrays, 8 × 15 k format (Agilent Technologies) were used. This microarray contained 5,069 different experimental probes spotted randomly in triplicate, representing 5,069 different gene clusters from Collembase [12,13]. The 60 nt long probes sequences were taken from a previous version of the microarray [14], and also contained manufacturer's control probes. All 8 microarrays used in this study were printed on the same glass slide. The design of this microarray (platform) is available from Gene Expression Omnibus [31] under accession number GPL7150.

### Microarray data analysis

Spot intensities were measured with Feature Extraction (9.1.3.1) Software (Agilent Technologies). Preprocessing, normalization, and differential gene expression assessment were all done in the limma [19] (2.14.5) package from the R (2.7.1) software environment [39]. This consisted of Edward's background correction [40], global loess normalization [41], and statistical analysis using linear models and empirical Bayes methods with subsequent multiple testing correction using Benjamini-Hochberg's method [20] (adjusted *p *< 0.05 was considered significant). Quality control was done by making MA-plots and box plots of each array; for each array the Expected LogRatio vs Observed LogRatio of the Agilent spike-in control probes were determined and they all showed an R^2 > 0.9. The assessment of differential expression of genes resulted in a mean log_2 _expression ratio (treated/untreated) and a *p*-value for each probe on the array. For hierarchical clustering mean normalized log_2 _expression ratios were used from each array, and was done in TIGR MEV version 3.1 [42], using Euclidean distance and average linkage. The raw and processed microarray data are available from the Gene Expression Omnibus [31] under accession number GSE14207. Annotation of the transcripts was performed using the BLAST [43] algorithm (e-value ≤ 1e-05). The sequence description in Additional file 2: Table S1 and Additional file 3: Table S2 are derived with Blasto2GO [44].

### Quantitative RT PCR

Primer sets were designed for 6 target genes (Fcc00508, Fcc00734, Fcc02784, Fcc03650, Fcc05793, Fcc05973) and one endogenous control gene *YWHAZ *(Fcc02512) with the software package Primer Express version 1.5 (Applied Biosystems) to have an annealing temperature of 60°C, and to amplify an amplicon of 80–120 base pairs (bp) with 45–55% GC content. PCR efficiency was determined by obtaining standard curves in triplicate for all primer sets with 4-fold dilutions of a standard batch cDNA. Primer sequences and PCR efficiency values are available in Additional file 5: Table S3. The RNA samples from the microarray experiment of the high phenanthrene (45.80 mg kg^-1^) concentration exposure were used for quantitative RT PCR (qPCR). Four biological replicates for both phenanthrene treated and reference were analyzed by qPCR. Approximately 1 μg input of total RNA per sample was used for reverse transcription using M-MLV reverse transcriptase (Promega) according to the manufacturer's protocol. The derived cDNA was diluted 1:5 and 2 μL was used in 20 μL PCR reaction volumes containing forward and reverse primers and Power SYBR Green PCR Master Mix (Applied Biosystems). qPCR reactions were performed in triplicate for each sample, on a DNA engine Opticon (MJ Research) using universal cycling conditions (10 min at 95°C; 15 s at 95°C, 1 min 60°C, 40 cycles). A mean normalized expression value (MNE) was calculated from the obtained Ct values with the Q-Gene module [45] using Fcc02512 (*YWHAZ*) as a reference gene for normalization of input cDNA.

### Statistical analysis

To verify that the phenanthrene concentrations used in this experiment had a significant effect on reproduction, a two tailed t-test (two-sample with unequal variance) was used in Microsoft Excel. The 50 genes that were differentially expressed in response to both phenanthrene concentrations were evaluated to identify those that significantly differed between the two concentrations. The log_2 _transformed normalized expression values, from each microarray, were also analyzed in a two tailed t-test (two-sample with unequal variance) in Microsoft Excel. To calculate a correlation between the microarray and qPCR platforms the Spearman's Rho correlation in SPSS (14.0 SPSS, Inc.) was performed on the mean log_2 _transformed normalized expression values.

## Abbreviations

ABC-transporter: ATP-binding cassette transporter; BH: Benjamini-Hochberg; BLAST: basic local alignment search tool; bp: base pairs; cDNA: complementary DNA; DNA: deoxyribonucleic acid; EC_10_: the effect concentration that inhibits reproduction by 10%; EC_50_: the effect concentration that inhibits reproduction by 50%; HPLC: high performance liquid chromatography; HSP: heat shock protein; ISO: International Organization for Standardization; loess: locally weighted scatterplot smoothing; LUFA: Landwirtschaftliche Untersuchungs- und Forschungsanstalt; MNE: mean normalized expression value; NADPH: nicotinamide adenosine dinucleotide phosphate (reduced form); nt: nucleotide; PAH: polycyclic aromatic hydrocarbon; qPCR: quantitative RT PCR; RNA: ribonucleic acid; ROS: reactive oxygen species; RT PCR: reverse transcription polymerase chain reaction; tRNA: transfer RNA; UDP: uridine diphosphate; VOMI: vitelline membrane outer layer protein I; VU: Vrije Universiteit.

## Authors' contributions

MB and BN performed the experiments and analyses of this research under supervision of DR. All authors were involved in the general set-up and design of the project. BN wrote the manuscript with contributions from all other authors. All authors read and approved the final manuscript.

## Supplementary Material

Additional File 1 **Figure S1: Venn diagram of significantly differentially expressed transcripts in response to phenanthrene**. In this diagram the amount of differential genes is shown for each phenanthrene concentration within the circles. The number in the right-bottom is the amount of non-significantly expressed genes. Click here for file

Additional File 2 **Table S1: All significantly differentially expressed transcripts in response to low concentration (24.95 mg kg^-1^) phenanthrene in soil**. This table contains all significantly differentially expressed transcripts and their log_2 _transformed fold change values. Click here for file

Additional File 3 **Table S2: All significantly differentially expressed transcripts in response to high concentration (45.80 mg kg^-1^) phenanthrene in soil**. This table contains all significantly differentially expressed transcripts and their log_2 _transformed fold change values. Click here for file

Additional File 4 **Figure S2: Heatmap for transcripts differentially expressed in response to the high concentration of phenanthrene (45.80 mg kg^-1^) and cadmium (57.9 mg kg^-1^) in soil**. Both concentrations represent the EC_50 _on reproduction after 28 days. Data of 4 microarrays were used for each xenobiotic exposure. Hierarchical clustering of log_2 _fold change values (treatment/reference) using Euclidean distance matrix, and average linkage. Red indicates upregulation and green downregulation compared to the reference control, black indicates no difference. The transcripts are named by their gene cluster in Collembase, followed by their putative function. Click here for file

Additional File 5 **Table S3: Oligos used in the qPCR analysis**. All sequences of the primers used in this study are shown, including PCR efficiency values. Click here for file

## References

[B1] Morillo E, Romero AS, Maqueda C, Madrid L, Ajmone-Marsan F, Grcman H, Davidson CM, Hursthouse AS, Villaverde J (2007). Soil pollution by PAHs in urban soils: a comparison of three European cities. J Environ Monitor.

[B2] Jongeneelen FJ (1992). Biological Exposure Limit for Occupational Exposure to Coal-Tar Pitch Volatiles at Cokeovens. Int Arch Occ Env Hea.

[B3] Baird WM, Hooven LA, Mahadevan B (2005). Carcinogenic polycyclic aromatic hydrocarbon-DNA adducts and mechanism of action. Environ Mol Mutagen.

[B4] Guengerich FP (2001). Common and uncommon cytochrome P450 reactions related to metabolism and chemical toxicity. Chem Res Toxicol.

[B5] Jakoby W, Ziegler D (1990). The enzymes of detoxication. J Biol Chem.

[B6] Homolya L, Varadi A, Sarkadi B (2003). Multidrug resistance-associated proteins: Export pumps for conjugates with glutathione, glucuronate or sulfate (Reprinted from Thiol Metabolism and Redox Regulation of Cellular Functions). Biofactors.

[B7] Fountain MT, Hopkin SP (2005). Folsomia candida (Collembola): A "standard" soil arthropod. Annu Rev Entomol.

[B8] Jensen J, Sverdrup LE (2003). Polycyclic aromatic hydrocarbon ecotoxicity data for developing soil quality criteria. Rev Environ Contam T.

[B9] (1999). Soil quality – Inhibition of reproduction of Collembola (*Folsomia candida*) by soil pollutants. ISO 11267.

[B10] Snape J, Maund S, Pickford D, Hutchinson T (2004). Ecotoxicogenomics: the challenge of integrating genomics into aquatic and terrestrial ecotoxicology. Aquat Toxicol.

[B11] van Straalen N, Roelofs D (2008). Genomics technology for assessing soil pollution. Journal of Biology.

[B12] Timmermans MJ, de Boer ME, Nota B, de Boer TE, Marien J, Klein-Lankhorst RM, van Straalen NM, Roelofs D (2007). Collembase: a repository for springtail genomics and soil quality assessment. Bmc Genomics.

[B13] Collembase. http://www.collembase.org/.

[B14] Nota B, Timmermans MJTN, Franken O, Montagne-Wajer K, Mariën J, Boer MEd, Boer TEd, Ylstra B, Straalen NMv, Roelofs D (2008). Gene Expression Analysis of Collembola in Cadmium Containing Soil. Environ Sci Technol.

[B15] Heckmann LH, Sibly RM, Connon R, Hooper HL, Hutchinson TH, Maund SJ, Hill CJ, Bouetard A, Callaghan A (2008). Systems biology meets stress ecology: linking molecular and organismal stress responses in Daphnia magna. Genome Biology.

[B16] Droge STJ, Paumen ML, Bleeker EAJ, Kraak MHS, van Gestelt CAM (2006). Chronic toxicity of polycyclic aromatic compounds to the springtail Folsomia candida and the enchytraeid Enchytraeus crypticus. Environ Toxicol Chem.

[B17] Crouau Y, Cazes L (2003). What causes variability in the Folsomia candida reproduction test?. Appl Soil Ecol.

[B18] Steibel JP, Rosa GJM (2005). On reference designs for microarray experiments. Statistical Applications in Genetics and Molecular Biology.

[B19] Smyth GK (2004). Linear models and empirical Bayes methods for assessing differential expression in microarray experiments. Statistical Applications in Genetics and Molecular Biology.

[B20] Benjamini Y, Hochberg Y (1995). Controlling the False Discovery Rate – a Practical and Powerful Approach to Multiple Testing. J Roy Stat Soc B Met.

[B21] Satoh T, Hosokawa M (2006). Structure, function and regulation of carboxylesterases. Chem-Biol Interact.

[B22] Kallberg Y, Oppermann U, Jornvall H, Persson B (2002). Short-chain dehydrogenases/reductases (SDRs) – Coenzyme-based functional assignments in completed genomes. Eur J Biochem.

[B23] Stroomberg GJ, Zappey H, Steen RJCA, van Gestel CAM, Ariese F, Velthorst NH, van Straalen NM (2004). PAH biotransformation in terrestrial invertebrates – a new phase II metabolite in isopods and springtails. Comp Biochem Phys C.

[B24] Ote M, Mita K, Kawasaki H, Daimon T, Kobayashi M, Shimada T (2005). Identification of molting fluid carboxypeptidase A (MF-CPA) in Bombyx mori. Comp Biochem Phys B.

[B25] Barbehenn RV, Stannard J (2004). Antioxidant defense of the midgut epithelium by the peritrophic envelope in caterpillars. J Insect Physiol.

[B26] Wiele T Van de, Vanhaecke L, Boeckaert C, Peru K, Headley J, Verstraete W, Siciliano S (2005). Human colon microbiota transform polycyclic aromatic hydrocarbons to estrogenic metabolites. Environ Health Persp.

[B27] Luscombe NM, Austin SE, Berman HM, Thornton JM (2000). An overview of the structures of protein-DNA complexes. Genome Biology.

[B28] Calow P (1991). Physiological costs of combating chemical toxicants: Ecological implications. Comparative Biochemistry and Physiology Part C: Comparative Pharmacology.

[B29] Franc NC, White K (2000). Innate recognition systems in insect immunity and development: new approaches in Drosophila. Microbes Infect.

[B30] Gong P, Guan X, Inouye L, Pirooznia M, Indest K, Athow R, Deng Y, Perkins E (2007). Toxicogenomic analysis provides new insights into molecular mechanisms of 2,4,6-trinitrotoluene in Eisenia fetida. Environ Sci Technol.

[B31] Gene Expression Omnibus. http://www.ncbi.nlm.nih.gov/geo/.

[B32] Shi LM, Reid LH, Jones WD, Shippy R, Warrington JA, Baker SC, Collins PJ, de Longueville F, Kawasaki ES, Lee KY (2006). The MicroArray Quality Control (MAQC) project shows inter- and intraplatform reproducibility of gene expression measurements. Nat Biotechnol.

[B33] Gasch AP, Spellman PT, Kao CM, Carmel-Harel O, Eisen MB, Storz G, Botstein D, Brown PO (2000). Genomic expression programs in the response of yeast cells to environmental changes. Mol Biol Cell.

[B34] de Boer ME, de Boer TE, Mariën J, Timmermans MJTN, Nota B, van Straalen NM, Ellers J, Roelofs D Reference genes for QRT-PCR tested under various stress conditions in Folsomia candida and Orchesella cincta (Insecta, Collembola). BMC Molecular Biology.

[B35] Wang YL, Barbacioru C, Hyland F, Xiao WM, Hunkapiller KL, Blake J, Chan F, Gonzalez C, Zhang L, Samaha RR (2006). Large scale real-time PCR validation on gene expression measurements from two commercial long-oligonucleotide microarrays. Bmc Genomics.

[B36] Gong P, Guan X, Inouye L, Deng Y, Pirooznia M, Perkins E (2008). Transcriptomic analysis of RDX and TNT interactive sublethal effects in the earthworm Eisenia fetida. Bmc Genomics.

[B37] Owen J, Hedley B, Svendsen C, Wren J, Jonker M, Hankard P, Lister L, Sturzenbaum S, Morgan A, Spurgeon D (2008). Transcriptome profiling of developmental and xenobiotic responses in a keystone animal, the oligochaete annelid Lumbricus rubellus. Bmc Genomics.

[B38] Svendsen C, Owen J, Kille P, Wren J, Jonker MJ, Headley BA, Morgan AJ, Blaxter M, Sturzenbaum SR, Hankard PK (2008). Comparative transcriptomic responses to chronic cadmium, flouranthene, and atrazine expousre in Lumbricus rubellus. Environmental Science & Technology.

[B39] The R Project for Statistical Computing. http://www.r-project.org/.

[B40] Edwards D (2003). Non-linear normalization and background correction in one-channel cDNA microarray studies. Bioinformatics.

[B41] Yang YH, Dudoit S, Luu P, Lin DM, Peng V, Ngai J, Speed TP (2002). Normalization for cDNA microarray data: a robust composite method addressing single and multiple slide systematic variation. Nucleic Acids Res.

[B42] Saeed AI, Sharov V, White J, Li J, Liang W, Bhagabati N, Braisted J, Klapa M, Currier T, Thiagarajan M (2003). TM4: A free, open-source system for microarray data management and analysis. Biotechniques.

[B43] Altschul SF, Madden TL, Schaffer AA, Zhang JH, Zhang Z, Miller W, Lipman DJ (1997). Gapped BLAST and PSI-BLAST: a new generation of protein database search programs. Nucleic Acids Res.

[B44] Conesa A, Gotz S, Garcia-Gomez JM, Terol J, Talon M, Robles M (2005). Blast2GO: a universal tool for annotation, visualization and analysis in functional genomics research. Bioinformatics.

[B45] Muller PY, Janovjak H, Miserez AR, Dobbie Z (2002). Processing of gene expression data generated by quantitative real-time RT-PCR. Biotechniques.

