# Supplementary material for: Transcriptomics reveals extensive inducible biotransformation in the soil-dwelling invertebrate Folsomia candida exposed to phenanthrene
Source: BMC Genomics. 2009 May 20;10:236. doi: 10.1186/1471-2164-10-236 (PMC2688526; doi:10.1186/1471-2164-10-236)

Figure S1: Venn diagram of significantly differentially expressed transcripts in response to phenanthrene. In this diagram the amount of differential genes is shown for each phenanthrene concentration within the circles. The number in the right-bottom is the amount of non-significantly expressed genes.


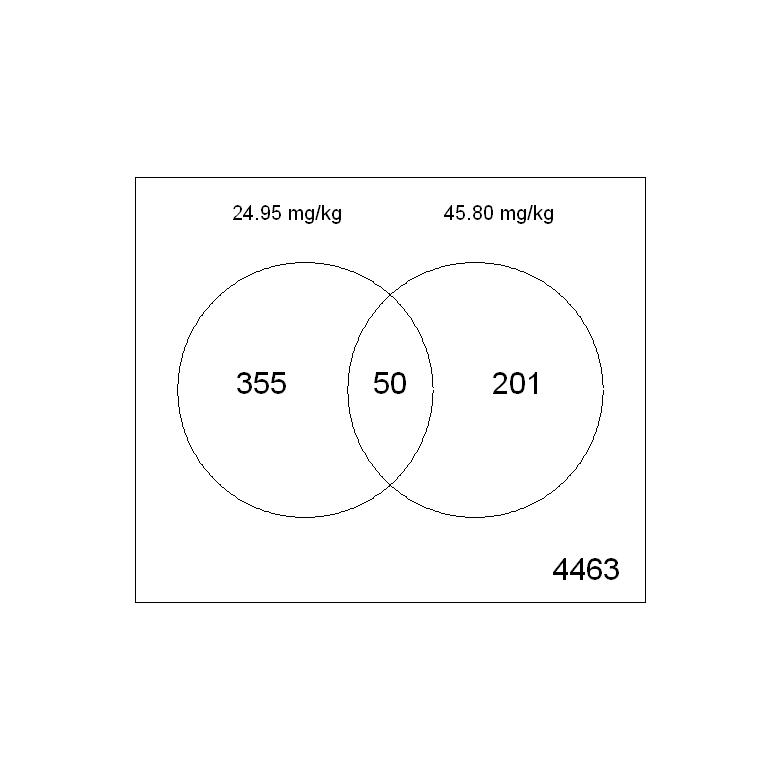

Supplement: Additional File 1 — Figure S1: Venn diagram of significantly differentially expressed transcripts in response to phenanthrene. In this diagram the amount of differential genes is shown for each phenanthrene concentration within the circles. The number in the right-bottom is the amount of non-significantly expressed genes. [file 1471-2164-10-236-S1.doc]
