# Supplementary material for: Transcriptomics reveals extensive inducible biotransformation in the soil-dwelling invertebrate Folsomia candida exposed to phenanthrene
Source: BMC Genomics. 2009 May 20;10:236. doi: 10.1186/1471-2164-10-236 (PMC2688526; doi:10.1186/1471-2164-10-236)

Figure S2: Heatmap for transcripts differentially expressed in response to the high concentration of phenanthrene (45.80 mg/kg) and cadmium (57.9 mg/kg) in soil. Both concentrations represent the EC_50_ on reproduction after 28 days. Data of 4 microarrays were used for each xenobiotic exposure. Hierarchical clustering of log_2_ fold change values (treatment/reference) using Euclidean distance matrix, and average linkage. Red indicates upregulation and green downregulation compared to the reference control, black indicates no difference. The transcripts are named by their gene cluster in Collembase, followed by their putative function.


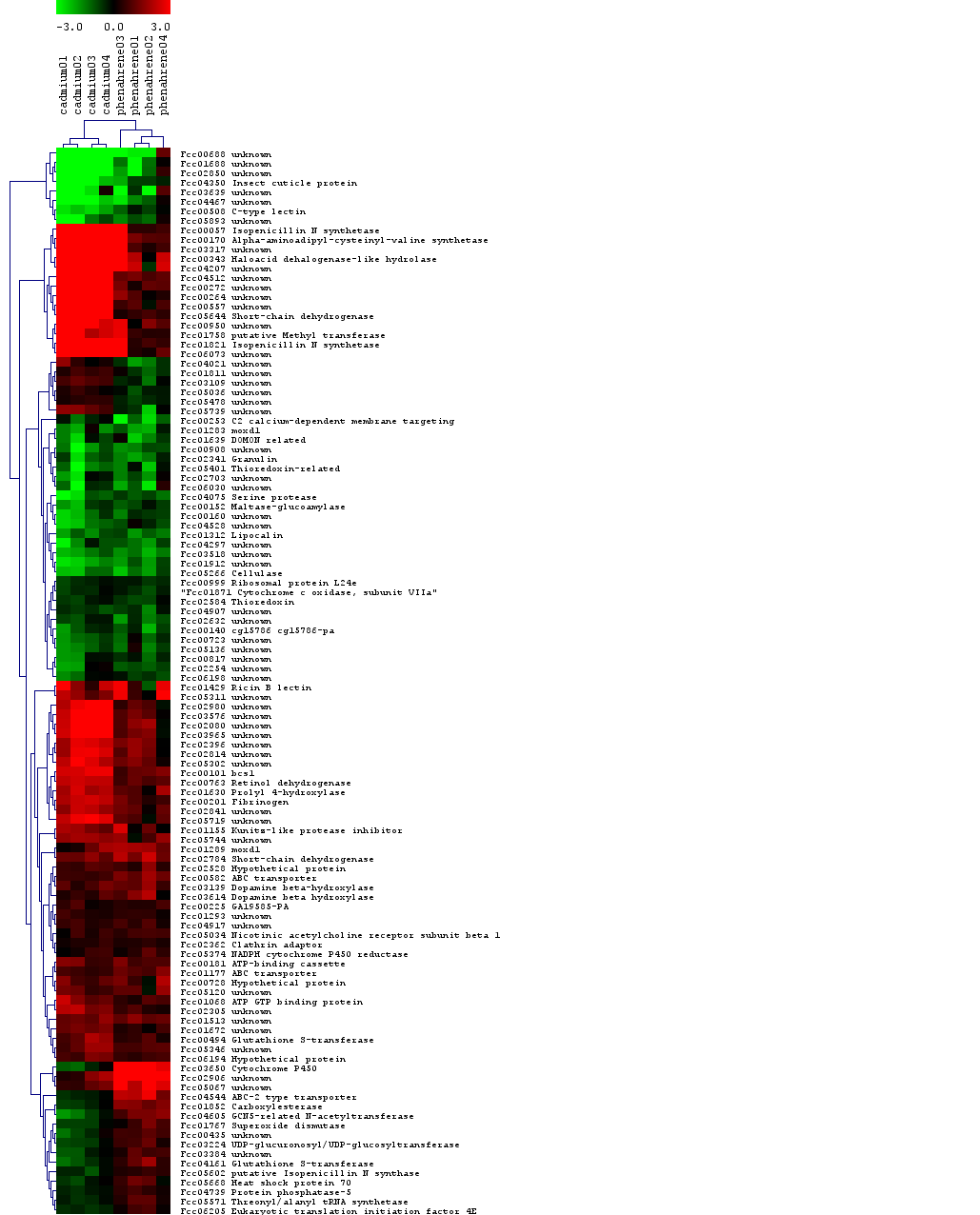

Supplement: Additional File 4 — Figure S2: Heatmap for transcripts differentially expressed in response to the high concentration of phenanthrene (45.80 mg kg-1) and cadmium (57.9 mg kg-1) in soil. Both concentrations represent the EC50 on reproduction after 28 days. Data of 4 microarrays were used for each xenobiotic exposure. Hierarchical clustering of log2 fold change values (treatment/reference) using Euclidean distance matrix, and average linkage. Red indicates upregulation and green downregulation compared to the reference control, black indicates no difference. The transcripts are named by their gene cluster in Collembase, followed by their putative function. [file 1471-2164-10-236-S4.doc]
