# Supplementary material for: Transcriptomics reveals extensive inducible biotransformation in the soil-dwelling invertebrate Folsomia candida exposed to phenanthrene
Source: BMC Genomics. 2009 May 20;10:236. doi: 10.1186/1471-2164-10-236 (PMC2688526; doi:10.1186/1471-2164-10-236)
Supplement: Additional File 5 — Table S3: Oligos used in the qPCR analysis. All sequences of the primers used in this study are shown, including PCR efficiency values. [file 1471-2164-10-236-S5.doc]

Table S3: Oligos used in the qPCR analysis.

| Cluster name | Putative description | Direction | Oligos sequence (5’-3’) | R^2^ | Efficiency |
| --- | --- | --- | --- | --- | --- |
| Fcc00508 | C-type lectin | Forward | TCATCCCTGCCGAACAGTATG | 0.932 | 2.39 |
|  |  | Reverse | TGCGAAGTGATAGACGCCATC |  |  |
| Fcc00734 | UDPGT | Forward | GACCGAAACTATGCCTGACCTTC | 0.996 | 2.00 |
|  |  | Reverse | ATATCCTGGCGTTCTTGTGGC |  |  |
| Fcc02512^*^ | YWHAZ | Forward | TCGCCCTCAACTTTTCCGTT | 0.995 | 2.10 |
|  |  | Reverse | TGCTATCGCTTCATCGAATGCT |  |  |
| Fcc02784 | Short-chain dehydrogenase | Forward | CAAAAATCTGCGAGGCAAGG | 0.993 | 2.20 |
|  |  | Reverse | CGCAGTTGCTTTCCCAATTC |  |  |
| Fcc03650 | Cytochrome P450 | Forward | AGCTTTGGATCCCCTCCAATT | 0.999 | 2.12 |
|  |  | Reverse | CGGTTTTGGTCGTGGCTAAAT |  |  |
| Fcc05793 | Hsp20 | Forward | GGAGGAAGTGAAGAGCTTGTTCAC | 0.972 | 1.81 |
|  |  | Reverse | TTGTTTTCCACCGAGATGGG |  |  |
| Fcc05973 | Glutathione S-transferase | Forward | TTTCAACATGCGAAGCATCG | 1.000 | 1.84 |
|  |  | Reverse | GTGTTCTGGCCACGTTTCTCTT |  |  |

^*^Fcc02512 (YWHAZ) is used as reference gene
